# Artificial Intelligence as a Catalyst for Value-Based Health Insurance in the United States: Narrative Review and Policy Perspective

**DOI:** 10.2196/84698

**Published:** 2026-03-20

**Authors:** Amol Kodan

**Affiliations:** 1 Department of Public Health, Monroe University, 434 Main StNew Rochelle, NY, 10801, United States, 1 646 393-8479

**Keywords:** artificial intelligence, AI, machine learning, ML, value-based care, VBC, health insurance, bundled payment, equity, accountable care organizations, transparency, Fee-For-Service, FFS

## Abstract

The United States health insurance system is at a critical crossroads. Inflating costs, fragmented care, and administrative inefficiencies have revealed the limitations of the Fee-for-Service (FFS) model. This long-standing structure, while once effective in expanding access, now struggles to deliver efficiency and value. Value-based care (VBC) aims to realign incentives toward outcomes, quality, and efficiency. This article explores how artificial intelligence (AI) can serve as the digital backbone to accelerate the transition from FFS to VBC. The article reviews evidence from bundled payment programs and Accountable Care Organizations (ACOs), examines AI-driven frameworks for cost prediction, outcome measurement, and risk adjustment, and discusses associated challenges and future considerations using an illustrative case. Bundled payment models, such as the Comprehensive Care for Joint Replacement program, have shown average savings of approximately $1012 per episode; whereas, the ACO REACH model achieved average savings of roughly $930 per beneficiary, relative to FFS benchmarks. AI applications provide scalable solutions for forecasting costs, optimizing care coordination, and supporting preventive interventions. A case vignette in congestive heart failure illustrates how AI-enabled VBC can reduce episode costs by approximately 20% under favorable implementation conditions. AI has the potential to accelerate the scaling of VBC by enhancing its efficiency, equity, and sustainability. However, realizing this promise requires safeguards for data quality, interoperability, fairness, and transparency. In the AI era, the defining measure of health insurance will shift from the number of claims processed to the number of lives improved.

## Introduction

Artificial intelligence (AI) is rapidly transforming the delivery and financing of health care, with applications ranging from clinical decision support to population health management. AI can play a key role in transitioning the traditional health insurance industry, which is standing at a decisive inflection point. Health insurance, a central pillar of health care services, is currently struggling with rising costs, fragmented care, uneven outcomes, and increasing administrative complexity. This highlights the inefficiencies of fee-for-service (FFS) models, where volume rather than value drives incentives [1,2]. In response, there is growing momentum toward value-based care (VBC), which prioritizes outcomes, quality, and efficiency over sheer quantity [3]. AI, through predictive modeling, data analytics, and natural language processing, provides the operational digital backbone that enables this shift to be both feasible and scalable [4]. By enhancing cost forecasting, strengthening care coordination, improving outcome measurement, and encouraging preventive interventions, AI creates the infrastructure for a system that rewards quality over quantity and aligns incentives with patient well-being.

As this article explores how artificial intelligence can accelerate the shift in US health insurance from traditional payment structures to modern outcome-oriented models, several key concepts are defined upfront to establish a clear foundation. The longstanding FFS model reimburses providers for each discrete service delivered, whereas VBC aligns payment with outcomes, quality, and efficiency. Accountable Care Organizations (ACOs) are provider networks jointly responsible for the total cost and quality of care for defined patient populations, and the Centers for Medicare & Medicaid Services (CMS) serves as the primary federal agency driving these reforms, including the forthcoming Transforming Episode Accountability Model (TEAM), a mandatory bundled payment initiative. Because AI integration relies on digital infrastructure, the manuscript briefly explains technical terms at first use, for example, cloud systems that continuously update data, automated pipelines that refresh when new clinical information arrives, and feedback systems, “closed-loop” models that allow AI to learn from the outcomes of prior recommendations. These clarifications ensure shared vocabulary for understanding the operational pathways through which AI can support scalable, equitable transitions from FFS to VBC. References to “real-time cloud architectures” denote cloud systems that continuously update information as new data arrives. At the same time, “event-driven pipelines” refer to automated data flows triggered whenever new clinical or claims data are generated. Likewise, “closed-loop architectures” refer to feedback systems in which AI-generated insights guide care decisions and the resulting outcomes are fed back into the model to improve future predictions. The term “high-throughput inference” refers to rapid processing that enables AI models to generate predictions at scale, and “interoperability standards” are the rules that ensure that different health IT systems can communicate with one another and reliably share their data.

This review argues that AI is not merely an adjunct tool but a structural enabler of scalable and accountable value-based insurance models (Textbox 1).

Textbox 1. **Key Takeaways**

- Today’s health care models require urgent reform, as nearly 25%‐30% of US health spending may represent avoidable waste.
- Value-based care (VBC) improves both quality and savings, with bundled payment programs reducing costs by about US $1012 per case.
- Artificial Intelligence (AI) can be harnessed to make value-based care scalable and efficient.
- Value-based care, particularly bundled episodes, will bring transparency to the opaque health care system.
- With responsible adoption, AI can act as a catalyst, transforming VBC from an aspiration into a reality by reducing costs and providing health care access to more people in the United States.
- Transparency, equity, fairness, and trust must form the foundation of AI-enabled health care.

Sources: [1,3,5]

This article is based on a narrative literature review conducted between January 2015 and December 2025. Relevant studies were identified through structured searches of PubMed, Google Scholar, and official reports from the CMS and other federal agencies. The review focused on real-world evaluations, program reports, and quantitative analyses examining the clinical, operational, and financial impacts of AI-enabled tools implemented in value-based care contexts. Sources were selected for relevance to the conceptual argument rather than through a systematic review protocol, with preference given to peer-reviewed publications, federal evaluations, and high-quality observational studies that reported measurable outcomes. No formal risk-of-bias assessment or meta-analysis was conducted, consistent with the narrative design.

Study identification, screening, and selection were performed by the author, with inclusion guided by relevance to the conceptual framework and the presence of reported outcome measures (eg, utilization, costs, or safety indicators). This review was not intended to be systematic or exhaustive; instead, it aimed to synthesize illustrative, policy-relevant evidence to support a conceptual analysis of AI as an operational enabler of value-based care.

## Value-Based Models: Bundled Payments and ACOs

Value-based care realigns incentives by tying reimbursement to outcomes, quality, and efficiency. In bundled payment models, all services associated with an episode of care (eg, hip or knee replacement) are combined into a single payment that covers diagnostics, procedures, hospitalization, rehabilitation, and follow-ups [5-11]. Complications or readmissions within a defined period (eg, 30‐90 d) remain the provider’s responsibility, thereby encouraging coordination across teams and settings [6,7].

## Evidence from the Comprehensive Care for Joint Replacement (CJR) Model Underscores the Impact:

**Figure 1.**
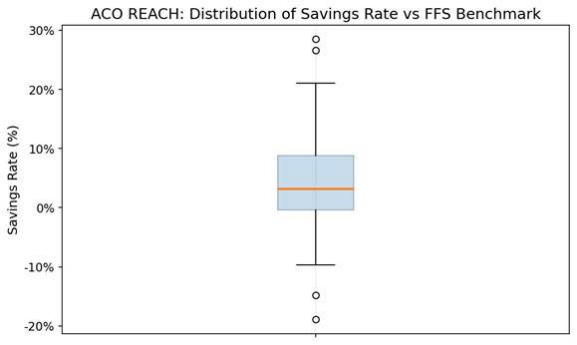
Average savings per beneficiary in the ACO REACH program compared with FFS benchmarks, 2022–2023 [8]. The boxplot shows the distribution of savings rates across participating organizations. The median savings (orange line) is approximately 3%‐4%, while the interquartile range (middle 50%) spans approximately 9%. Most ACOs cluster within this range, indicating modest but consistent positive savings relative to FFS. ACO: accountable care organizations; FFS: fee-for-service.

In performance year 2021‐2022, Care for Joint Replacement (CJR) hospitals reduced episode payments by an average of $1012 per joint replacement and $1171 per elective replacement compared with FFS hospitals, while maintaining quality outcomes [5,10]. This is illustrated in Multimedia Appendix 1. Accountable Care Organizations (ACOs) extend the reach of value-based incentives by aligning care delivery across entire patient populations. An ACO network brings together physicians, hospitals, and other health care providers, who work collaboratively to deliver coordinated, high-quality care for a defined patient population. Their goal is to improve patient outcomes while reducing any unnecessary costs. This arrangement shifts the focus from volume to value. Unlike an FFS arrangement, ACOs assume responsibility for the total cost and quality of care across primary, specialty, and hospital services [8]. When expenditure remains below benchmarks and metrics are met, providers share the savings; when spending exceeds benchmarks, they may share the losses. CMS data from ACO REACH indicate average savings of approximately $930 per beneficiary relative to FFS benchmarks, indicating broad efficiency gains under population-level accountability [8,9] (Figure 1).

Together, evidence from bundled payment models shows that value-based care can reduce costs while maintaining quality. Episode-based accountability (eg, CJR) and population-level accountability (eg, ACOs) each demonstrate efficiency gains for VBC by aligning incentives with coordination and outcomes, thereby providing a foundation for AI-enabled tools to enhance scalability and equity further [5,8,11].

**Figure 2.**
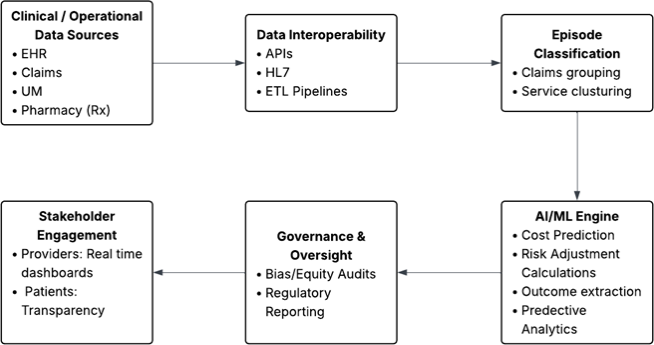
Illustration of how artificial intelligence (AI) works as a catalyst in the transition from FFS to VBC: Workflow from data capture to predictive insights. API: application programming interface; AI: artificial intelligence; EHR: electronic health record; ETL: extract–transform–load; FFS: fee-for-service; HL7: Health Level Seven; ML: machine learning; VBC: value-based care; UM: utilization management.

Policy momentum continues to build. CMS has finalized the TEAM, a mandatory bundled payment initiative scheduled to begin in 2026 across selected metropolitan areas, signaling a sustained federal commitment to value-based reimbursement [11]. This convergence sets the stage for examining how AI can accelerate the transition to scalable, equitable, value-based care. Figure 2 illustrates how AI acts as a catalyst in the shift towards VBC.

## Discussion

### Value-Based Care Initiatives

Value-based care initiatives, including bundled payments and ACOs, have shown promise in reducing costs and improving outcomes [3,7]. Still, widespread adoption and scaling up these models require a digital foundation capable of integrating heterogeneous data, enabling seamless coordination, and supporting continuous improvement. Artificial intelligence integrates clinical and claims data, applying machine learning and predictive analytics to generate actionable feedback at the point of care [4,12]. By doing so, AI can operationally enable value-based care from a policy concept into a practical reality.

### Data Capture and Real-Time Interoperability

The starting point for the workflow is the entry of structured and unstructured data from clinical and operational systems, electronic health records (EHRs), laboratory and imaging results, claims streams, pharmacy transactions, and admission, discharge, and transfer events [13,14]. Instead of static captures or retrospective batch uploads, modern architectures use APIs (Application Programming Interfaces) and event-driven pipelines to stream provisional claims, vitals, and medication updates into a real-time, unified cloud environment. The cloud system provides elastic compute and storage, as well as large-scale data engineering and high-throughput inference, ensuring that predictive models are continuously updated as new information becomes available. This cloud-based infrastructure enables predictive models to be constantly updated as new data are added and processed.

### Episode Classification and Benchmark Pricing

Once data is standardized and stored in the cloud environment, encounters are grouped into clinically coherent bundles using AI-assisted methods. Automated groupers use medical codes (ICD, International Classification of Diseases/CPT, Current Procedural Terminology) along with unsupervised clustering of EHR events to define episode boundaries accurately. Automated systems use codes and patterns in health records to categorize related care into a single episode, such as for heart failure or any surgery. These episode definitions serve as the basis for benchmarking. Benchmark pricing sets the baseline for comparing patient-specific predictions, enabling early detection of deviations and facilitating timely intervention. These groupings are then compared with past data to set expected costs. This benchmark makes it easier to spot unusual changes early and take timely action.

### AI/ML Engines and the Closed-Loop Feedback Cycle

At the core of this architecture, various machine learning and AI turn raw inputs into predictive insights. Machine learning models (eg, gradient boosting and recurrent neural networks) estimate episode costs relative to expected benchmarks and dynamically recalculate them as new utilization signals accumulate. Survival analysis models, such as DeepSurv, along with recurrent neural networks, generate continuously updated readmission probabilities by leveraging longitudinal patient histories and population-level patterns. Additional forecasting tools simulate potential care pathways after discharge and analyze the likelihood of emergency visits or nursing facility stays. Reinforcement learning agents simulate postacute utilization pathways, projecting probabilities of emergency department visits or skilled nursing placement under different discharge strategies. Transparency tools identify the main factors, such as medication use or laboratory markers, that drive these risks or costs. Explainability frameworks, such as SHAP, provide transparency by surfacing the most influential measures, including medication adherence or biomarker trajectories, which drive cost or risk predictions.

These insights are not confined to data reports; they are delivered directly into provider workflows via electronic health records, dashboards, and alerts. They are embedded directly into workflows through clinical decision support webhooks in the EHR, role-specific dashboards, and automated alerts. Care teams can immediately adjust treatment plans, discharge timing, or follow-up as needed. The results of these actions are ingested back into the system, enabling the models to learn and improve, while oversight mechanisms ensure fairness, compliance, and transparency. This closed-loop architecture ensures models continuously improve while governance layers enforce fairness, compliance, and interpretability.

#### Illustrative Case Vignette

Let us review a potential case of AI-enabled value-based care in congestive heart failure, which would cost $40,000 using the fee-for-service model. Consider a patient admitted with congestive heart failure. Detection begins when claims data are processed by an automated grouper that maps CPT and ICD codes and links them to related EHR encounters. A real-time pipeline streams discharge summaries, laboratory results, vital signs, reconciliation notes, and provisional claims into a cloud-based system. A cost-prediction engine estimated the total spending for this episode at $38,000, compared with the episode benchmark of $34,000. This provides early warning to providers that the case is more likely to be complicated, as the engine has predicted a higher episode cost than the benchmark for similar episodes. The model continually adjusted itself as new laboratory results and service utilization information became available. It used a hybrid of gradient-boosting and recurrent models, along with a readmission risk model, to integrate longitudinal predictors, including prior admissions, BNP (B-type natriuretic peptide) trends, and adherence patterns, and to estimate a high 30-day readmission risk. A reinforcement-learning forecaster simulates discharge options and flags an increased likelihood of acute-care utilization when cardiology follow-up is delayed.

Model explainability tools (eg, SHAP, Shapley Additive exPlanations) highlight renal impairment, elevated BNP, and incomplete medication optimization as the main cost drivers. These insights are surfaced in real-time to the provider team, prompting the deployment of remote monitoring and expedited follow-up care. As a result, deterioration is prevented, readmission is avoided, and the episode cost falls to $32,500. Outcomes then feed back into the learning system, improving future predictive accuracy.

While the vignette demonstrates how predictive modeling, explainability tools, and closed-loop feedback could theoretically reduce utilization and episode costs, these outcomes should not be interpreted as universally replicable or guaranteed in real-world settings. Actual performance varies significantly across patient populations, provider capabilities, data infrastructure, and local care-delivery environments. Consequently, the example should be viewed as a conceptual model that highlights operational possibilities rather than a validated, generalizable result.

### AI as a Force Multiplier for VBC

This case demonstrates that value-based care, through bundled payments and coordinated services, can reduce waste and enhance efficiency. With the adoption of AI, the use of real-time data pipelines, large-scale cloud integration, and predictive modeling significantly increases the impact. Costs are reduced not only by avoiding unnecessary utilization but also by dynamically tailoring interventions to individualized patient trajectories, guided by transparent, predictive analytics. The result is a delivery and financing system that achieves superior outcomes at lower cost, proving that AI is not just a reporting tool but the operational backbone that makes value-based care scalable, sustainable, and clinically impactful across the health care ecosystem [4].

#### Early Evidence From the Real-World Studies

Collectively, these studies suggest early signals of clinical and financial benefit, though heterogeneity limits generalizability. Emerging real-world deployments demonstrate meaningful reductions in readmissions, mortality, preventable harm, and episode costs. Table 1 summarizes selected real-world evaluations. The current evidence base for AI-enabled value-based care remains early and uneven. Most published results derive from retrospective analyses, pilot implementations, or single-system deployments rather than large-scale, prospective studies across heterogeneous populations. To establish the actual clinical and financial value of AI within bundled payments, ACOs, and Medicare Advantage models, the next phase of research must focus on robust real-world evidence: multisite prospective evaluations, payer-provider collaborative trials, and federally supported demonstration projects designed to measure equity, cost-effectiveness, and long-term patient outcomes. Such rigorous empirical work is essential not only to validate current promising signals but also to guide scalable, trustworthy, and accountable adoption of AI across diverse care environments.

**Table 1.** Real-world evaluation studies.

Study/setting	AI^a^ intervention	Value-based or performance context	Clinical outcomes	Financial outcomes / ROI^b^
Bennett et al, 2025 (Safety-Net Health System)^c^ [15]	EHR-integrated^d^ discharge checklist, AI readmission-risk model, population-health dashboard for heart failure	Pay-for-performance contracts tied to 30-day readmission metrics	30-day readmission rate declined from 27.9% to 23.9% (*P*<.004) across rolling monthly cohorts; racial equity gap in readmissions eliminated; all-cause mortality reduced (HR 0.82, 95% CI 0.68‐0.99)	Retained US $7.2 million in at-risk funding following an approximately US $1 million investment (ROI>7:1)
Burdick et al, 2020 (9 US hospitals) [16]	ML^e^-based sepsis early-warning algorithm	Hospitals participating in quality-linked reimbursement programs	Among patients admitted with sepsis (n=17,758 encounters), in-hospital mortality decreased by 39.5%, hospital length of stay decreased by 32.3%, and 30-day readmissions decreased by 22.7% (*P*<.001 each)	Cost reductions inferred through avoided clinical deterioration and shorter hospital stays (no direct dollar estimates reported)
Alanazi et al., 2023 (ICU cohort) [17]	ML-based sepsis prediction model using EHR laboratory and vital-sign streams	Evaluated for alignment with quality and outcome monitoring in critical care	Improved model performance for early sepsis prediction in ICU^f^ patients; the study focused on prediction accuracy and feature importance rather than downstream clinical outcomes	Potential for reduced ICU length of stay and utilization costs discussed conceptually; no empirical cost outcomes reported
Ericson et al., 2022 (Cost-effectiveness evaluation) [12]	Commercial AI sepsis early-warning system (NAVOY® Sepsis)	Modeled for suitability in value-oriented payment environments	Health-economic modeling projected reduced complications and mortality with earlier sepsis detection (approximately 3 h earlier recognition)	Modeled net savings of approximately €1,009 per ICU patient and favorable incremental cost-effectiveness ratios, often dominant under conventional willingness-to-pay thresholds
Epelde, 2024 (Hospital benchmarking study with perspective overview) [18]	AI-supported patient safety and clinical deterioration alerts	Linked to hospital quality-improvement and patient-safety benchmarking metrics	Synthesized evidence showing associations between AI-supported alerts and improved patient-safety indicators across hospital benchmarks	Reported economic gains attributed to reduced preventable harm and operational efficiency improvements (benchmarking synthesis)

a AI: artificial intelligence.

b ROI: return on investment.

c For Bennett et al, outcomes were reported as time-series rates over the evaluation period rather than a single fixed cohort; therefore, absolute denominators are not presented.

d EHR: electronic health record.

e ML:machine learning.

f ICU: intensive care unit.

### Challenges and Considerations

While AI has immense potential to reinforce and scale up the foundation of a value-based framework, shaping the future, we must be both optimistically cautious and prepared to tackle the challenges and obstacles ahead [15-17]. Achieving this vision requires robust safeguards to ensure the responsible and equitable adoption. Data silos and inconsistent and incomplete records can weaken predictive accuracy. Without proper oversight, poorly designed algorithms, or insufficient transparency and regulation, bias may persist. Building and maintaining stakeholder trust will be vital to ensure equitable implementation.

Governance frameworks, such as explainability tools [for example, SHAP and Local Interpretable Model-agnostic Explanations (LIME), together with model validation and equity audits, help ensure that predictions remain interpretable, unbiased, and clinically trustworthy [19]. Insights generated by these systems are delivered to stakeholders in an actionable manner. For example, providers gain dashboards and decision-support tools integrated into workflows, while patients receive greater visibility into outcomes and costs, fostering trust in the value-based care ecosystem.

Despite these safeguards, challenges may persist. Another significant barrier is the digital divide, in which smaller providers, who face infrastructure gaps, experience substantial setbacks and may face implementation barriers. Smaller payers and providers often lack the IT infrastructure or machine learning operations capacity to implement AI at scale, raising concerns that only large organizations may benefit. Bridging these structural gaps and broadening provider participation will be critical for equitable adoption nationwide.

Another obstacle lies in patient trust and engagement. As AI becomes more deeply embedded in reimbursement and care delivery, patients may fear opaque decision-making or automated claim denials. Recent lawsuits over insurer use of algorithms have already underscored these risks, highlighting the importance of transparency and accountability in protecting patient confidence. Regulatory clarity regarding algorithmic accountability and liability will also be essential.

To meaningfully bridge the operational and regulatory gaps that limit AI adoption- particularly among smaller, rural, or resource-constrained providers- the discussion must expand beyond identifying barriers. It must also outline actionable pathways forward. Several strategies can support more equitable implementation. First, federal and state agencies could incentivize the development of shared cloud-based infrastructures or AI “utility platforms” that reduce the financial and technical burden on small practices while maintaining strong governance standards. Second, standardized data ontologies and open APIs mandated across EHR vendors would mitigate data fragmentation and promote interoperability, lowering the entry cost for providers lacking advanced informatics capabilities. Third, payers and CMS could integrate AI adoption incentives directly into value-based contracts, such as by offering technical assistance, subsidized analytics tools, or grant-based funding for digital modernization. Finally, multistakeholder collaboratives, by bringing together large health systems, payers, academic partners, and small community practices, can support knowledge transfer and reduce disparities in readiness. These policy mechanisms would allow AI-enabled VBC innovations to diffuse more evenly, preventing a widening digital divide and ensuring that smaller providers are active beneficiaries rather than casualties of the technology transition.

## Conclusion—A Way Ahead

As we move toward a new era, the most significant shift on the horizon is a paradigm shift where the objective measure of health insurance will no longer be the number of claims processed, but the number of lives improved. As we pave the way for a healthier future, it is essential to align insurers, providers, regulators, and technologists around a common framework of equity, transparency, and accountability. As we adopt a broader range of technologies and AI in the health care insurance sector, let us build the foundational blocks of transparency, equity, and efficiency. The transformation from volume to value in US health insurance, which was once aspirational, is now a tangible reality, driven by policy reforms and enabled by AI.

AI will not “fix” the limitations of health insurance on its own, but, if used appropriately, can serve as a transformative enabler of this shift. If grounded in fairness and trust, AI-enabled value-based care can transform health care financing from a source of friction into a driver of healthier populations, where every life is valuable and matters. Success will depend not only on adoption but also on equity, transparency, and trust.

## Supplementary material

10.2196/84698 Multimedia Appendix 1 Comprehensive Care for Joint Replacement (CJR) bundled payment impact on episode payments (Centers for Medicare & Medicaid Services, CMS performance year 6, 2021‐22)

## Abbreviations

ACO: Accountable Care Organizations
AI: artificial intelligence
BNP: B-type natriuretic peptide
CJR: Comprehensive Care for Joint Replacement
CMS: Centers for Medicare & Medicaid Services
CPT: current procedural terminology
FFS: fee-for-service
SHAP: Shapley Additive exPlanations
TEAM: Transforming Episode Accountability Model
VBC: value-based care
