# Supplementary material for: Artificial Intelligence as a Catalyst for Value-Based Health Insurance in the United States: Narrative Review and Policy Perspective
Source: JMIR AI. 2026 Mar 20;5:e84698. doi: 10.2196/84698 (PMC13004588; doi:10.2196/84698)
Supplement: Multimedia Appendix 1 [file ai-v5-e84698-s001.docx]

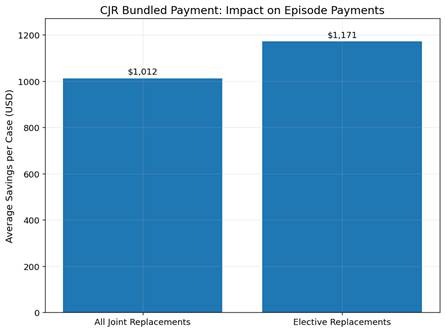


Figure S1. Comprehensive Care for Joint Replacement (CJR) bundled payment impact on episode payments [(Centers for Medicare & Medicaid Services, CMS Performance Year 6, 2021–‐22]). Average episode payment per joint replacement, expressed in USD per episode, comparing hospitals participating in the CJR Model with those under fee-for-service (FFS) benchmarks. Data represent CMS Innovation Center evaluation results for Performance Year 6 (2021–‐2022). The model showed mean savings of approximately $1,012 per episode for all joint replacements and $1,171 for elective replacements, with no loss in quality outcomes.
